# Investigation of the key chemical structures involved in the anticancer activity of disulfiram in A549 non-small cell lung cancer cell line

**DOI:** 10.1186/s12885-018-4617-x

**Published:** 2018-07-21

**Authors:** Kate Butcher, Vinodh Kannappan, Rajagopal Sharada Kilari, Mark R. Morris, Christopher McConville, Angel L. Armesilla, Weiguang Wang

**Affiliations:** 1 Faculty of Science & Engineering, University of Wolverhampton, Wolverhampton, WV1 1LY UK; 2 School of Pharmacy, University of Birmingham, Birmingham, UK

**Keywords:** Disulfiram, Cancer stem cells, Copper, Non-small cell lung cancer, Reactive oxygen species, Diethyldithiocarbamate, S-methyl-diethyldithiocarbamate

## Abstract

**Background:**

Disulfiram (DS), an antialcoholism medicine, demonstrated strong anticancer activity in the laboratory but did not show promising results in clinical trials. The anticancer activity of DS is copper dependent. The reaction of DS and copper generates reactive oxygen species (ROS). After oral administration in the clinic, DS is enriched and quickly metabolised in the liver. The associated change of chemical structure may make the metabolites of DS lose its copper-chelating ability and disable their anticancer activity. The anticancer chemical structure of DS is still largely unknown. Elucidation of the relationship between the key chemical structure of DS and its anticancer activity will enable us to modify DS and speed its translation into cancer therapeutics.

**Methods:**

The cytotoxicity, extracellular ROS activity, apoptotic effect of DS, DDC and their analogues on cancer cells and cancer stem cells were examined in vitro by MTT assay, western blot, extracellular ROS assay and sphere-reforming assay.

**Results:**

Intact thiol groups are essential for the in vitro cytotoxicity of DS. S-methylated diethyldithiocarbamate (S-Me-DDC), one of the major metabolites of DS in liver, completely lost its in vitro anticancer activity. In vitro cytotoxicity of DS was also abolished when its thiuram structure was destroyed. In contrast, modification of the ethyl groups in DS had no significant influence on its anticancer activity.

**Conclusions:**

The thiol groups and thiuram structure are indispensable for the anticancer activity of DS. The liver enrichment and metabolism may be the major obstruction for application of DS in cancer treatment. A delivery system to protect the thiol groups and development of novel soluble copper-DDC compound may pave the path for translation of DS into cancer therapeutics.

**Electronic supplementary material:**

The online version of this article (10.1186/s12885-018-4617-x) contains supplementary material, which is available to authorized users.

## Background

Due to the time and cost for new drug development [1], drug repositioning has become an attractive strategy in recent years for anticancer drug development [2]. Disulfiram (DS) specifically inhibits aldehyde dehydrogenase (ALDH) and blocks the further degradation of acetaldehyde coverted from alcohol. The cummulation of acetaldehyde causes an unpleasent effect which makes DS one of the first line anti-alcoholism drugs [3] that has been used in clinic for almost 70 years. In the last three decades, it was reported that DS has excellent in vitro anticancer activity in a wide range of cancer cell lines [4–15]. DS inhibits proteasome/NFκB pathway [5, 16], MDR1 [17], topoisomerase, MMP [18], NPL4 [15] and manipulates MAP kinase pathways [13]. It eradicates cancer stem cells (CSCs) and significantly reverses chemoresistance in resistant cancer cell lines [9–11, 13]. Its cytotoxicity in cancer cells is copper dependent [8, 9, 19]. Although DS shows high in vitro toxicity in cancer cells, there was almost no positive clinical data published in cancer patients (https://clinicaltrials.gov/ct2/results?term=disulfiram+AND+cancer&Search=Search). Therefore, elucidating the discord between the anticancer activity of DS in laboratory and clinic is of significant clinical importance in cancer treatment.

**Fig. 1:**
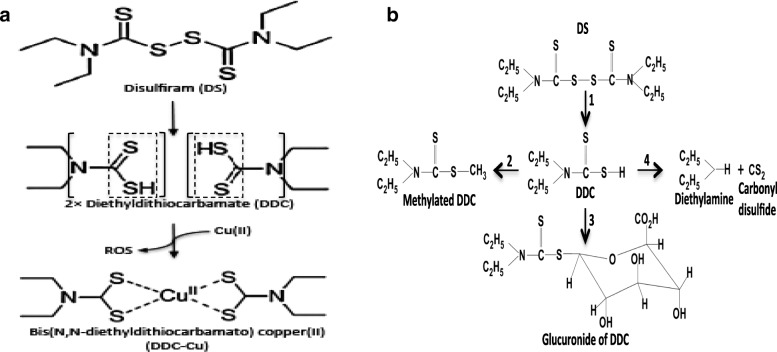
Biotransformation and reaction of DS with copper in humans. **a** Biotransformation of DS. DS is promptly reduced to DDC in the bloodstream by glutathione reductase system of erythrocytes (1). In liver, DDC is methylated by S-methyltransferase to form S-Me-DDC (2), glucuronidated by glucuronyl transferase to form glucuronidated DDC (3) and non-enzymatic degradation into diethylamine and carbonyl disulfide (4). **b** Chelation of DS with copper. The DDC derived from DS chelates Cu(II) to form Cu(DDC)_2_ and generates ROS. The thiol group (dashed box) is essential for the reaction. The solid frame highlights thiuram structure

In serum, DS is rapidly reduced to form two molecules of diethyldithiocarbamate (DDC). DDC is a very strong chelator of transition divalent metal ions, mainly copper(II) (Cu) and zinc (Fig. 1a) [4, 8, 9, 20–23]. The data from our and other groups demonstrate that the in vitro cytotoxicity of DS is copper-dependent [4, 9]. In serum-free medium without copper supplement, DS completely loses its cytotoxicity in cancer cell lines [13]. Cu plays a crucial role in redox reactions. When DDC contacts Cu, the chelating reaction between them triggers the generation of reactive oxygen species (ROS) [19, 24], which damage DNA, protein and lipids leading to cancer cell death. ROS are extremely transient species with very short lifetime due to their high chemical reactivity and can only penetrate very short distance in tissues [25]. To target cancer cells, the reaction of DS and Cu must take place inside or adjacent to cancer cells. In addition to ROS generated from DDC and Cu reaction, bio(N,N-diethyldithiocarbamato) copper(II) (Cu-DDC), the end product derived from DDC and Cu reaction, is also cytotoxic in cancer cells. [19] Our previous work indicates that there are two phases of cytotoxic effect of DS on cancer cells, the instant damages induced by the reaction between DS and Cu and the delayed killing caused by the end product, Cu-DDC [19]. The sulfhydryl groups in DDC (dashed box in Fig. 1a) are indispensible for the chelating reaction between DDC and Cu and formation of Cu-DDC complex. After oral administration, DS is immediately reduced to form DDC in the gastrointestinal system and the bloodstream of the portal vein. DDC is then enriched in the liver and promptly enzymatically converted to S-methyl-DDC (S-Me-DDC) and gluconidated DDC by S-methyl-transferase and glucuronyl transferase, respectively, or completely degraded to diethylamine and carbonyl disulfide (Fig. 1b). In the liver, the S-Me-DDC is oxidized by microsomal oxidative metabolism to form diethylthiocarbamic acid methyl ester (Me-DETC) and S-methyl N,N-diethythiolcarbamate sulforxide (MeSO-DETC) [26]. The Me-DETC and MeSO-DETC are the functional units for inhibition of ALDH in hepatocytes [26, 27]. So, DS remains the antialcoholism activity after oral administration. All of these DS metabolites lose their functional thiol groups for chelation of Cu. This might compromise the anticancer efficacy of DS when orally administered in cancer patients. Therefore enrichment and metabolism of DS in the liver becomes the bottleneck for translation of DS into cancer treatment. To overcome these limitations, we recently developed nano-encapsulated DS, e.g. liposomal- and PLGA-DS, which are intravenously injectable [11, 14]. The nano-encapsulation protects the thiol groups in DS and extends its half-life in the serum from less than 2 min to over 7 h and successfully delivers the intact DS to cancer tissues [14]. In combination with oral administration of copper gluconate, the nano-encapsulated DS demonstrated significantly stronger anticancer efficacy in mouse breast, liver, ovarian, lung and brain cancer models [11, 14, 28–31] (and our unpublished data).

DS is a small molecule with a molecular weight of 296.4 Da consisting of sulfhydryl groups (dashed box in Fig. 1a), thiuram structure (brackets in Fig. 1a) and the ethyl groups. It has been suggested that the sulfhydryl groups in DDC are essential for the cytotoxicity of DS in cancer cells [19, 24]. The role of the thiuram structure and ethyl groups in the cytotoxicity of DS in cancer cells is still not clear. Elucidation of the relationship between the chemical structure and anticancer activity of DS will be helpful for modification of DS and development of novel lead compounds for further drug development.

In this study, we examined the in vitro anticancer activity of several DS and DDC related compounds. Our results indicate that the intact sulfhydryl groups and the thiuram structure are critical for maintaining the anticancer activity of DS and DDC. In contrast, modification of the ethyl groups had no significant effect on their anticancer activity.

## Methods

### Cell line and reagents

The non-small cell lung cancer (NSCLC) A549 (CCL-185) and H23 (CRL-5800) cell lines were purchased from ATCC (Middlesex, UK). Copper chloride (CuCl_2_, Cu), disulfiram (DS), diethyldithiocarbamate (DDC), S-methyl-N,N-diethyldithiocarbamate (S-Me-DDC), tetramethylthiuram disulfide (TMDS), 2-hydroxy-dithiobenzoic acid (HDTA), 4-imidazoledithiocarboxylic acid (IDTA), 2,4,6-Trimercaptotriazine (TMT) and poly-2-hydroxyethyl methacrylate (poly-HEMA) were purchased from Sigma (Dorset, UK). Bis(N,N-diethyldithiocarbamate)-copper(II) (Cu-DDC) was from Santa Cruz (Dallas, TX, USA). Annexin V kit was from Roche Applied Sciences (Burgess Hill, UK). Fc OxyBURST Assay Reagents was purchased from Invitrogen, Molecular Probes (Waltham, MA, USA). ALDEFLUOR kit was from StemCell Tech (Durham, NC, USA). FITC mouse Anti-Human CD44 from BD Biosciences (Oxford, UK).

### Cell culture and cytotoxicity analysis

The A549 and H23 cells were cultured in DMEM (Lonza, Wokingham, UK) supplemented with 10% FCS, 2 mM L-glutamine, 50 units/ml penicillin, 50 μg/ml streptomycin. For in vitro cytotoxicity assay, the cells (5000/well) were cultured in 96-well flat-bottomed microtiter plates overnight and exposed to different compounds with or without CuCl_2_ (10 μM) for 72 h, then subjected to a standard MTT assay [32].

### *In vitro* spheroid culture and cytotoxicity assay

To culture the spheres, cells were cultured in poly-HEMA coated ultra-low adherence flasks or plates to prevent cell adhesion. The spheres were cultured, at a density of 20,000 cells/ml, in stem cell culture medium [SCM: serum-free DMEM-F12 (Lonza) supplemented with B27 (Invitrogen, Paisley, UK), 20 ng/ml epidermal growth factor (EGF, Sigma), 10 ng/ml basic fibroblasts growth factor (b-FGF, R & D System, Abingdon, UK), 10 μg/ml insulin, 20 μg/ml heparin, 45% D-glucose, 1% L-glutamine, 1% penicillin, streptomycin, amphotericin mix (Sigma)]. After 7 days culture, the spheres were trypsinised. The dispersed cells were exposed to different drugs at the indicated concentration for 6 h and subjected to ALDH and CD44 analysis. For in vitro sphere reformation assay, drug-treated cells were resuspended in drug-free SCM at a density of 1 × 10^5^ cells/ml and seeded in ultra-low adherence 24-well plates and cultured for further 7 days.

#### Detection of ALDH positive population

The ALDH positive population was detected by ALDEFLUOR kit (StemCell Tech., Durham, NC, USA) following the supplier’s instruction. The cells (2.5 × 10^5^) were exposed to different drugs for 6 h and were analyzed after incubation in ALDH substrate containing assay buffer for 30 min at 37 °C. The specificity was determined by exposure to diethylaminobenzaldehyde (DEAB, 30μM), a specific ALDH inhibitor.

#### Flow cytometric analysis of CD44 expression

The adherent or sphere cells were trypsinised and the cells (2.5 × 10^5^) were incubated with CD44 antibodies (BD Pharmingen, Oxford, UK) for 30 min at 4 °C. The cells (20,000 events) were examined within 1 h after staining on a BD Facscalibur.

### Western blotting analysis

The protein expression levels were determined by staining with primary antibodies and HRP conjugated secondary (1:5000, Armersham, Buckinghamshire, UK) antibody. The cleaved PARP, BCL2, BAX (1:1000, Abcam, Cambridge, UK) and anti-α-tubulin (1:8000, Sigma) primary antibodies were diluted in 5% fat-free milk-TBST. The signal was detected using an ECL Western blotting detection kit (GeneFlow, Staffordshire, UK).

### Measurement of extracellular ROS activity

The extracellular ROS levels were determined using Fc OxyBURST® Assay Reagents (ThermoFisher Sci., Paisley, UK) following the supplier’s instruction. Briefly, OxyBURST Green was diluted in H_2_O at a final concentration of 1μg/ml and 100μl was added into each well of a black 96-well plate. The compounds and CuCl_2_ (10μl of each at 10μM concentration) were added into each well. The H_2_O and H_2_O_2_ (10μl of 1:100 diluted) were used as negative and positive control, respectively. N-acetyl-L-cysteine (NAC, 2μl of 100 mM stock solution) was used as ROS inhibitor to confirm ROS activity. Immediately, the oxidative product release in the reaction was detected by a continuous fluorescence increase excited at 492 and emission of 520 nm at integration of 1 s. The rate of fluorescence increase was proportional to the amount of oxidative species generated.

### Assessment of apoptosis by Annexin-V/PI assay

Apoptotic status was determined by FITC-conjugated Annexin-V/PI assay kit (Roche) using flow cytometry following the manufacturer’s instructions. Briefly, 2 × 10^5^ cells were seeded in 6 well flat bottom plates for 24 h and exposed to drugs for 16 h. Dead cells were collected and the remaining cells were rinsed with PBS and detached using trypsin. Detached cells were resuspended in 100 μl binding buffer containing FITC-conjugated Annexin-V (10 mg/mL)/PI (50 mg/ml) and incubated at RT for 15 min. The cells were diluted in 400 μl of PBS and analyzed by a FACScan flow cytometry (Becton Dickinson, Franklin Lakes, NJ USA). Apoptosis and necrosis were evaluated using FL2 (PI) vs FL1 (Annexin V) plots. The cells stained with Annexin V only were classified as early apoptosis and the Annexin V and PI double-stained cells were classified as late apoptosis or necrosis.

### Statistical analysis

SPSS 13.0 Student’s *t* test and one-way analysis of variance (ANOVA) followed by Tukey’s Multiple Comparison Test were used to calculate the differences. Data were expressed as mean ± SD. *P* ≤ 0.05 was considered as significantly change.

## Results

### Intact sulfhydryl groups are indispensible for the cytotoxicity of DS and DDC but the ethyl groups can be modified

**Fig. 2:**
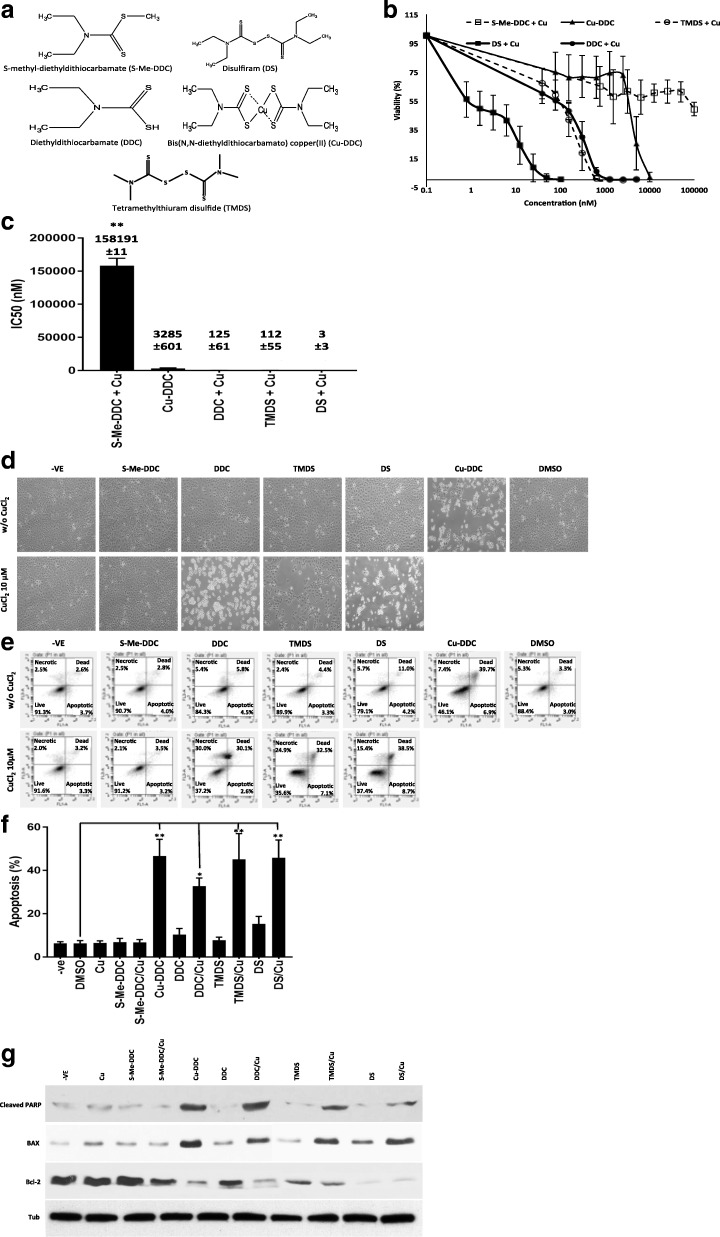
Cytotoxic effect of DS and related compounds on A549 NSCLC cell line. **a** The chemical structures of DS-related compounds used in this study. **b** Viability curves of A549 cells in different treatments. The cytotoxicity of different compounds in monolayer-cultured A549 cells was examined. All the treatments except Cu-DDC were supplemented with CuCl2 (in a consistent concentration of 10μM). The cells were dosed for 72 h and then subjected to MTT assay. **c** IC_50_ (nM) values. **d** The morphology of the cells subjected to different treatments. The cells were treated with S-Me-DDC (10 μM), Cu-DDC (5μM), DDC (1μM), TMDS (1μM), DS (1μM) with or without Cu (10 μM) for 6 h and then release in drug-free medium overnight. The microscopic images were taken at 40× magnification. **e** and **f** Annexin V analysis of apoptosis. The cells were exposed to different compounds at the above concentrations with or without 10 μM of CuCl_2_ for 6 h and immediately subjected to Annexin V analysis. **g** Western blotting detection of the expression of apoptosis related proteins. The cells were subjected the same treatments as D before western blotting analysis. *N* = 3, * *p* < 0.05, ** *p* < 0.01

First, cytotoxicity of five analogues of DS and DDC (Fig. 2a) in A549 cells was compared. We used S-Me-DDC, in which the thiol group is methylated, to determine the importance of thiol group in cytotoxicity of DS and DDC. We also changed the ethyl groups to methyl groups in DDC to examine the role of the ethyl groups in the cytotoxicity of DDC. The cytotoxicity of these modified DS and DDC was compared with DS, DDC and Cu-DDC. MTT cytotoxicity assay demonstrated that methylation of the thiol group in DDC abolishes the cytotoxicity of DDC in Cu-containing medium (Fig. 2b and c). The IC_50_ of S-Me-DDC plus Cu (158,191 nM) is 1250 times higher than that of DDC plus Cu (125 nM) and also significantly higher than those of other compounds (3 to 3285 nM). In combination with Cu, the TMDS, in which the ethyl groups in DS are replaced with methyl groups, remains highly toxic in A549 cells. The IC_50_ of TMDS/Cu (112 nM) is still significantly higher than that of DS/Cu (3 nM)(*p* < 0.01). In comparison with Cu-DDC and DDC/Cu, TMDS/Cu, DS/Cu are more toxic in A549 cells. The similar effect of these compounds was also observed in another NSCLC cell line, H23 (Additional file 1). The morphology of cells after different treatments is showed in Fig. 2d. DDC, DS, TMDS in combination with Cu and Cu-DDC induce cancer cell apoptosis. The apoptotic effect was completely blocked when the thiol group is methylated (Fig. 2e and f). In line with previous reports [4, 13], the apoptotic effect of DDC, DS and TMDS is copper-dependent. The apoptotic results were also confirmed by western blotting analysis of the expression of some apoptosis-related proteins, e.g. cleaved PARP, BCL2 and BAX (Fig. 2g).

### The intact thiol groups are essential for targeting CSC-like cells

**Fig. 3:**
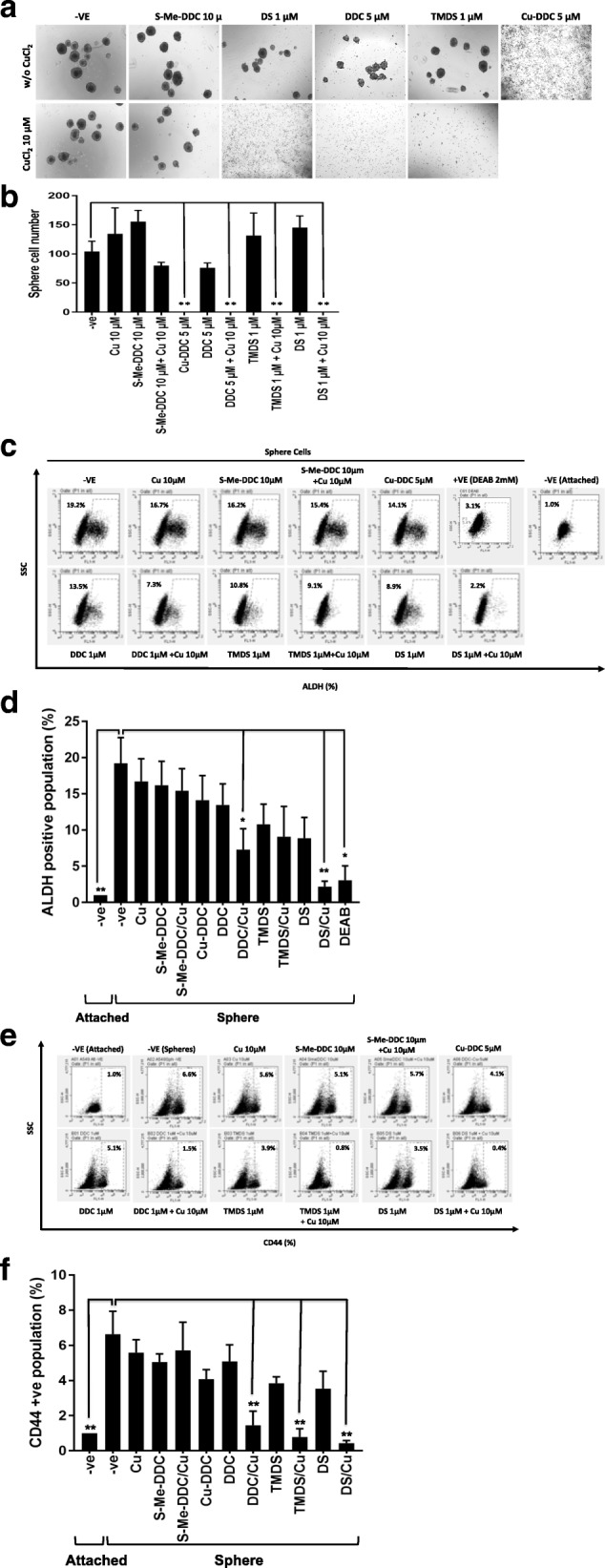
The effect of different compounds on CSC population in A549 cell line. **a** and **b** Sphere-reformation assay. The A549 cells formed spheres after cultured in stem cell medium for 7 days. The spheres were exposed to S-Me-DDC (10 μM), Cu-DDC (5 μM), DDC (1 μM), TMDS (1 μM) and DS (1 μM) in combination with or without CuCl_2_ (10 μM) for 6 h. The spheres were trypsinized and cultured in drug free SCM at a density of 5000 cells/well in ultralow attached 24-well plates for another 7 days. The sphere numbers in each well were counted. **c** and **d** The effect of different treatments on ALDH activity in sphere cells. The spheres were trypsinized and exposed to different treatments for 6 h before ALDEFLUOR analysis. **e** and **f** The effect of different treatments on CD44 expression in sphere cells. *n* = 3, ** < 0.01

Our previous studies indicate that DS very strongly targets CSC-like cells. In this study, we further examined if the structure modification also influences the effect of DS on CSC-like cells. Figure 3a and b show the effect of different compounds on cancer cell sphere reformation ability. The results demonstrate that the inhibiting effect of DDC on sphere reformation was abolished by the methylation of the thiol group in S-Me-DDC. In contrast, the cytotoxic effect of DS on CSC-like cells was not affected by replacement of the ethyl with methyl groups. Furthermore, the effect of different compounds on the expression of CSC markers was examined. In line with the inhibiting effect on sphere reformation, S-Me-DDC/Cu lost its inhibiting effect on ALDH activity. DDC and DS, in combination with Cu, significantly inhibit the ALDH activity in sphere cells (Fig. 3c and d). TMDS also inhibited ALDH activity although it did not reach statistical significance. Cu-DDC blocks the sphere reformation and inhibits ALDH activity, to a lesser extent. We also examined the effect of these compounds on CD44, another CSC marker. In combination with Cu, DS, TMDS and DDC significantly inhibited the expression of CD44. Cu-DDC and DS alone also inhibited CD44 to a lesser extent (Fig. 3e and f). S-methylated DDC completely lost the inhibiting effect on CD44 expression. This data suggests that the CSC targeting effect of DS also depends on the intact thiol group in DS and DDC. Modification of ethyl groups has no influence on the anti-CSC activity of DS.

### Functional thiol groups are responsible for extracellular ROS generation

**Fig. 4:**
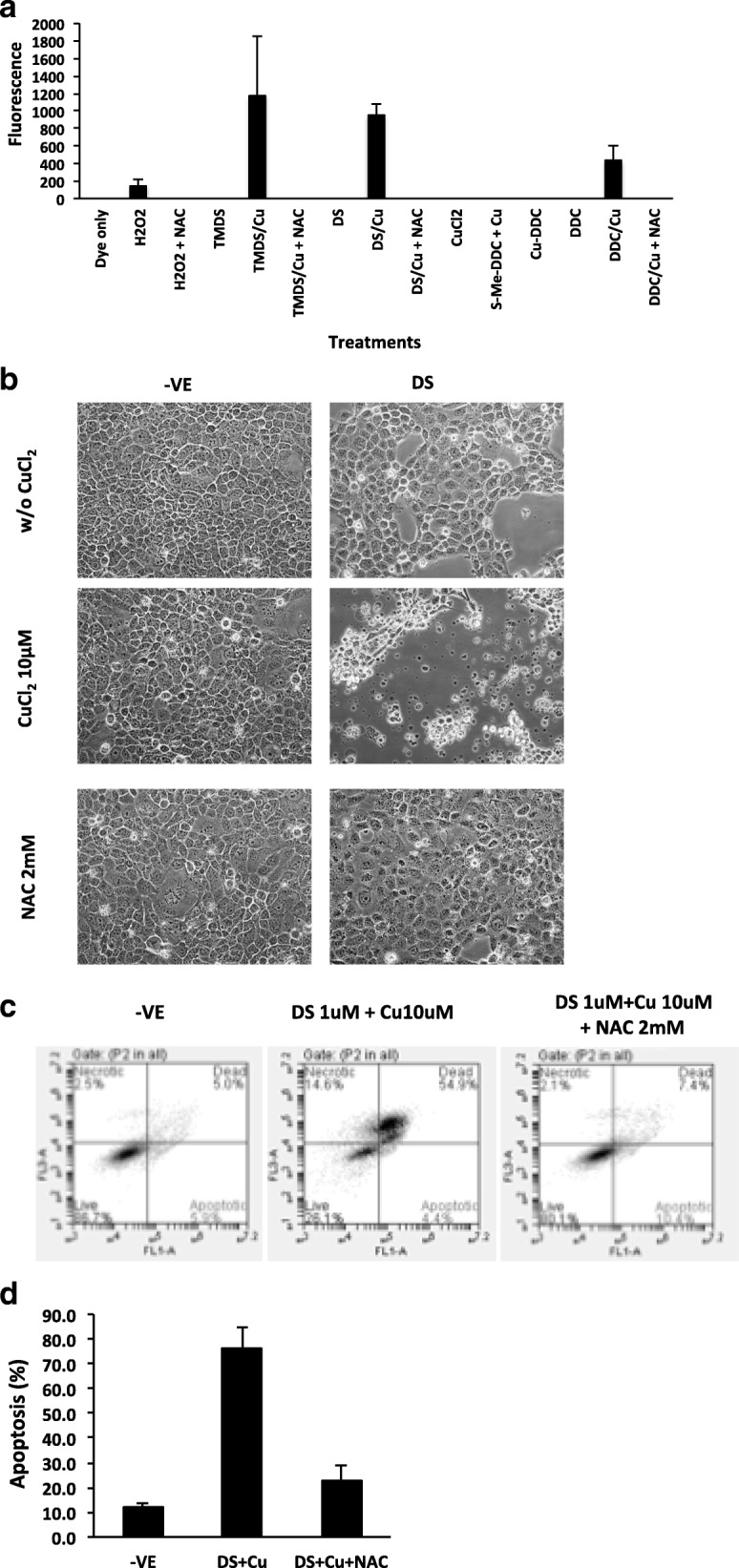
Copper chelation generated ROS was responsible for cytotoxicity. **a** ROS activity generated from different compounds (10 μM) in combination with or without CuCl_2_ (10 μM). H2O2 (1:100 diluted) and dye only were positive and negative control respectively. **b** The cytotoxicity of DS was reversed by NAC. The cells were exposed to DS (1 μM) and CuCl_2_ (10 μM) with or without NAC (2 mM) for 6 h and released in drug-free medium. **c** and **d** The apoptosis induced by DS was reversed by NAC. The cells were exposed to DS (1 μM) and CuCl_2_ (10 μM) with or without NAC (2 mM) for 6 h and subjected Annexin V analysis

We previously demonstrated that the chelation of DS and Cu generated ROS extracellularly [19]. In line with our previous result, DS and DDC reacted with Cu and generated ROS in cell free medium. Methylation of the thiol group (S-Me-DDC) completely blocked ROS generation. In contrast, replacement of the ethyl groups with methyl groups (TMDS + Cu) had no affect on ROS activity. The ROS generation was reversed by addition of NAC into the reaction (Fig. 4a). Using DS as a model, we examined the reversing effect of NAC on DS and Cu induced cell death and apoptosis. Figure 4b to d show that the DS and Cu induced cell death and apoptosis were reversed by addition of NAC into the culture medium. It indicates that the cytotoxicity of DS and DDC in A549 cells was induced, at least partly, by the ROS generated from chelating reaction between DS, DDC and Cu.

### The thiuram structure is also essential for the cytotoxicity of DS

**Fig. 5:**
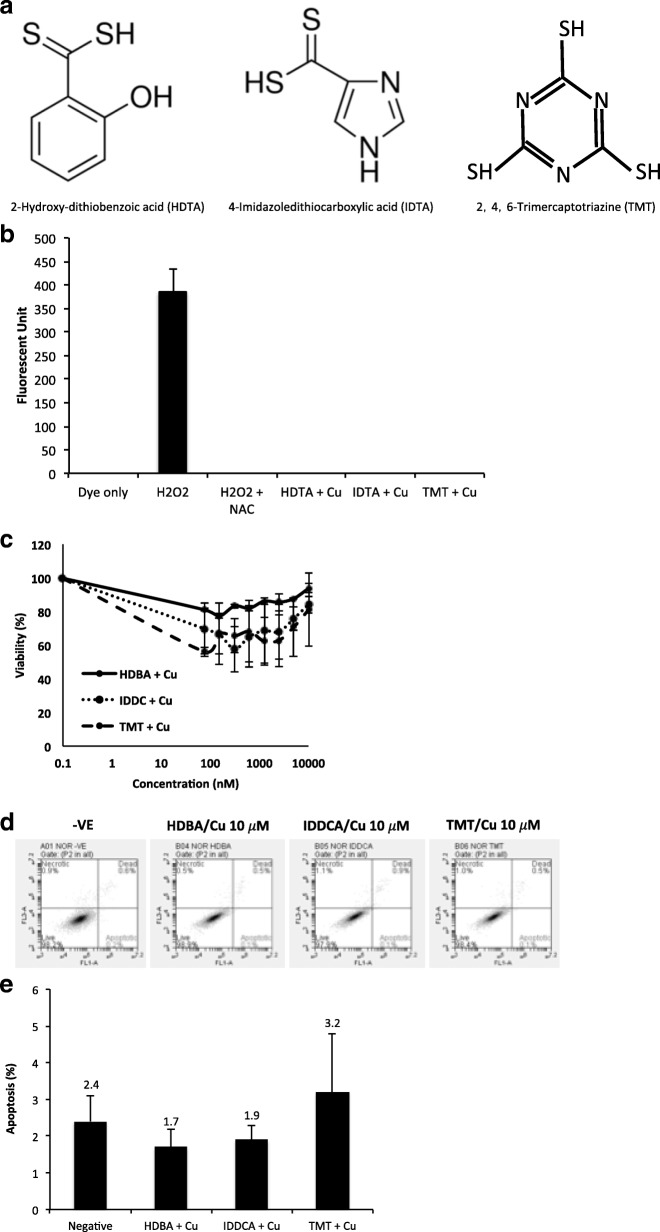
Thiuram structure is essential for anticancer activity of DS. **a** Chemical structures of thiol group containing compounds without thiuram structure. **b** Cytotoxicity of TMT, HDTA and IDTA on A549 cells. The cells were exposed to the compounds in combination with CuCl_2_ (10 μM) for 72 h and subjected to MTT assay. **c** and **d** Apoptotic status of A549 cells exposed to 10 μM of TMT, HDTA, IDTA in combination with CuCl_2_ (10 μM). The cells were treated with the above chemicals for 6 h and then subjected to Annexin V analysis. **e** Extracellular ROS detection. ROS activity generated from the mixture of TMT, HDTA, IDTA (10 μM of each) and CuCl_2_ (10 μM) was measured. H_2_O_2_ (1:100) was used as a positive control

The above experiments confirmed the indispensible role of the thiol groups in the cytotoxicity of DS. Furthermore, we modified the DDC chemical structure and examine the influence of thiuram structure on the anticancer activity of DS. For this purpose, we replaced the nitrogen in DDC with a nitrogen-containing five-membered heterocycle (4-Imidazoledithiocarboxylic acid, IDTA) or a phenol (2-Hydroxy-dithiobenzoic acid, HDTA). The thiol groups are intact in these two compounds but the thiuram structure is disrupted by replacing nitrogen with carbon (Fig. 5a). Because chelation of copper by thiol groups in DS or DDC generates ROS and is responsible for the cytotoxic effect, we also tested a six-membered heterocycle with three thiol groups (2, 4, 6-Trimercaptotriazine), a very strong chelator of divalent metal ions, including Cu [33]. The cytotoxicity of these compounds was examined by MTT assay in combination with 10 μM of CuCl_2_. Although these compounds can chelate Cu, no ROS was detected when mixed with Cu (Fig. 5b). Cytotoxic effect was not detected by MTT assay when these compounds were co-cultured with Cu (Fig. 5c and Table 1). Addition of TMT, IDTA and HDTA with Cu into the culture medium did not induce apoptosis (Fig. 5d and e). These results indicated that in addition to the thiol group, the thiuram structure in DS and DDC is also essential for the cytotoxicity in cancer cells. The nitrogen atom and its position in the chemical structure of DS and DDC are critical for the cytotoxicity of DS and DDC to cancer cells.

**Table 1:** Cytotoxicity of disulfiram related compounds in A549 cell line

	IC_50_ (μM)
TMT + Cu	> 10
HDTA + Cu	> 10
IDTA + Cu	> 10

## Discussion

DS shows very strong anticancer activity in laboratory but not in clinic. Elucidation of the key anticancer chemical structure of DS will speed up its translation into clinic as a cancer treatment [34]. The anticancer activity of DS is Cu(II) and other transition metal ions dependent. The thiol group is essential for the chelating reaction between DDC and Cu. After oral administration, DS is rapidly reduced in vivo to DDC by plasma glutathione reductase [35] and albumin [36]. DDC is catalyzed by cytochrome p450 in liver to form S-Me-DDC and then further oxidized to Me-DETC and MeSO-DETC [26, 37] which are the active forms of DS with antialcoholism activity [26]. In this study, we examined the in vitro cytotoxicity of S-Me-DDC, the major metabolites of DS in the liver. In combination with Cu, DS and DDC are highly toxic to cancer cells and target CSC-like cells. In contrast, S-methylation completely reverses the cytotoxicity of DDC and abolishes its anti-CSC activity. This result indicates that the methylation of DS in liver may be responsible for the poor response of cancer patients to orally administered DS. This may explain the discrepancy of anticancer-efficacy between clinic and laboratory. In order to translate DS from an anti-alcoholism application into a cancer treatment, protection of the thiol group is essential. To this end, we developed intravenously injectable nanoparticle-encapsulated DS to protect the thiol group. In combination with copper, the nano-encapsulated DS has demonstrated very strong anticancer efficacy in mouse models [11, 14]. In contrast, substitution of the ethyl groups with methyl groups did not significantly affect the cytotoxicity. In combination with Cu, TMDS blocked sphere-reformation and inhibited CSC traits in lung cancer cell line. This observation is consistent with the previous report that the ethyl groups substituents have no influence on the reaction of metal chelation [38]. The copper-dependent proteasome-inhibitory and apoptosis-inducing activity of DS was not affected by replacing the ethyl groups with pyrrolidine, morpholine and piperazine [39, 40]. The ethyl groups and their substitution determine the hydrophilicity of the compounds. TMDS is more hydrophilic than DS and thus it has less ability to penetrate into cancer cells. This explains why TMDS is less toxic (Fig. 2b and c). Similar result were observed when the ethyl groups were substituted with a pyrrolidine ring attached with a hydrophilic hydroxyl group [39].

Cu-DDC is also toxic to lung cancer cells and targets CSC-like cells. Copper, a redox metal ion, can produce ROS using Fenton and Haber Weiss reactions and induce apoptosis [34]. Development of copper-based drugs has been very attractive for anticancer drug development [41, 42] but transport of copper into cells is strictly regulated by a trans-membrane copper transporter Ctr1 [5]. Cu-DDC is highly lipophilic and can bypass the copper transporter system to penetrate into cells freely [4]. Decomposition of DDC inside cells may release the chelated copper, which will generate oxidative stress, resulting in damage to DNA, RNA and protein. The Cu-DDC induced intracellular ROS activity has been reported in our previous studies [10, 13]. When the cells were treated with DS in medium containing CuCl_2_, the intracellular copper concentration was rapidly increased 8-fold [4]. Both the intracellular Cu uptake and DS induced toxicity was blocked by co-incubation with bathocuproine disulfonic acid, a non-membrane-permeable Cu chelator. A recent report suggests that Cu-DDC is a major functional anticancer unit of DS [15]. In comparison with DS and DDC, Cu-DDC is more stable in the bloodstream [43] and easier to develop as a new copper-based anticancer drug.

We also examined the role of the thiuram structure (Fig. 1a) on the anticancer activity of DS and DDC. In HDTA and IDTA, the nitrogen in DDC is replaced by hydroxyl benzene and imidazole respectively, while TMT has intact thiol groups. All of these compounds are very strong copper chelators. The copper complex generated by the reaction is highly hydrophobic and can easily penetrate into cells. There was no extracellular ROS generated when these compounds were mixed with Cu (Fig. 5b) and no cytotoxicity or apoptosis was observed when the cells were cultured at the high concentration of these compounds (10 μM) and Cu. Our results indicated that along with the thiol groups, the thiuram structure is also essential for the anticancer activity of DS and DDC.

## Conclusions

The high and selective anticancer activity of DS in the laboratory has been known for more than three decades but no promising clinic data were published. We hypothesized that the discrepancy between laboratory and clinic was introduced by the metabolism of DS in cancer patients. The anticancer activity is dependent on ROS generated from the reaction between DS and copper and the final product, Cu-DDC. When orally administered, DS is enriched in liver and promptly metabolized to S-methyl and S-glucuronide DDC. The S-methylation and glucuronidation block the chelation of DS with copper and compromise its anticancer activity although the antialcoholism function remained. In this study, we first demonstrated that the S-methy-DDC, one of the major metabolites of DS in liver, had no cytotoxicity in cancer cells. Our study also suggested that the thiuram structure in the DS is also indispensible for its anticancer activity. In contrast, modification of the ethyl groups in DS did not significantly affect its cytotoxicity in cancer cells. The findings in this study indicates that a delivery system to protect the thiol groups in DS or development of a soluble Cu-DDC formulation will pave the path for translation of DS into cancer therapeutics.

## Additional file

Additional file 1: Cytotoxic effect of DS and related compounds on H23 NSCLC cell line. A. (PDF 8177 kb)

## Abbreviations

ALDH: Aldehyde dehydrogenase
CSCs: Cancer stem cells
Cu: Copper(II)
Cu-DDC: Bio(N,N-diethyldithiocarbamato) copper(II)
DDC: Diethyldithiocarbamate
DS: Disulfiram
Me-DETC: Diethylthiocarbamic acid methyl ester
MeSO-DETC: S-methyl N,N-diethythiolcarbamate sulforxide.
ROS: Reactive oxygen species
S-Me-DDC: S-methyl- diethyldithiocarbamate

## References

[1] Ashburn TT, Thor KB (2004). Drug repositioning: identifying and developing new uses for existing drugs. Nat Rev Drug Discov.

[2] Nosengo N (2016). Can you teach old drugs new tricks?. Nature.

[3] Eneanya DI, Bianchine JR, Duran DO, Andresen BD (1981). The actions of metabolic fate of disulfiram. Annu Rev Pharmacol Toxicol.

[4] Cen D, Brayton D, Shahandeh B, Meyskens FL, Farmer PJ (2004). Disulfiram facilitates intracellular cu uptake and induces apoptosis in human melanoma cells. J Med Chem.

[5] Chen D, Cui QC, Yang H, Dou QP (2006). Disulfiram, a clinically used anti-alcoholism drug and copper-binding agent, induces apoptotic cell death in breast cancer cultures and xenografts via inhibition of the proteasome activity. Cancer Res.

[6] Guo X, Xu B, Pandey S, Goessl E, Brown J, Armesilla AL, Darling JL, Wang W (2010). Disulfiram/copper complex inhibiting NFkappaB activity and potentiating cytotoxic effect of gemcitabine on colon and breast cancer cell lines. Cancer Lett.

[7] Marikovsky M, Nevo N, Vadai E, Harris-Cerruti C (2002). Cu/Zn superoxide dismutase plays a role in angiogenesis. Int J Cancer.

[8] Iljin K, Ketola K, Vainio P, Halonen P, Kohonen P, Fey V, Grafstrom RC, Perala M, Kallioniemi O (2009). High-throughput cell-based screening of 4910 known drugs and drug-like small molecules identifies disulfiram as an inhibitor of prostate cancer cell growth. Clin Cancer Res.

[9] Liu P, Brown S, Goktug T, Channathodiyil P, Kannappan V, Hugnot JP, Guichet PO, Bian X, Armesilla AL, Darling JL (2012). Cytotoxic effect of disulfiram/copper on human glioblastoma cell lines and ALDH-positive cancer-stem-like cells. Br J Cancer.

[10] Liu P, Kumar IS, Brown S, Kannappan V, Tawari PE, Tang JZ, Jiang W, Armesilla AL, Darling JL, Wang W. Disulfiram targets cancer stem-like cells and reverses resistance and cross-resistance in acquired paclitaxel-resistant triple-negative breast cancer cells. Br J Cancer. 2013;109:1876–85. 10.1038/bjc.2013.534 PMC3790184 24008666

[11] Liu P, Wang Z, Brown S, Kannappan V, Tawari PE, Jiang J, Irache JM, Tang JZ, Armesilla AL, Darling JL (2014). Liposome encapsulated disulfiram inhibits NFκB pathway and targets breast cancer stem cells in vitro and in vivo. Oncotarget.

[12] Wang W, McLeod HL, Cassidy J (2003). Disulfiram-mediated inhibition of NF-kappaB activity enhances cytotoxicity of 5-fluorouracil in human colorectal cancer cell lines. Int J Cancer.

[13] Yip NC, Fombon IS, Liu P, Brown S, Kannappan V, Armesilla AL, Xu B, Cassidy J, Darling JL, Wang W (2011). Disulfiram modulated ROS-MAPK and NFkB pathways and targeted breast cancer cells with cancer stem cell like properties. Br J Cancer.

[14] Wang Z, Tan J, McConville C, Kannappan V, Tawari PE, Brown J, Ding J, Armesilla AL, Irache JM, Mei QB (2017). Poly lactic-co-glycolic acid controlled delivery of disulfiram to target liver cancer stem-like cells. Nanomedicine.

[15] Skrott Z, Mistrik M, Andersen KK, Friis S, Majera D, Gursky J, Ozdian T, Bartkova J, Turi Z, Moudry P (2017). Alcohol-abuse drug disulfiram targets cancer via p97 segregase adaptor NPL4. Nature.

[16] Cvek B, Dvorak Z (2008). The value of proteasome inhibition in cancer. Can the old drug, disulfiram, have a bright new future as a novel proteasome inhibitor?. Drug Discov Today.

[17] Loo TW, Clarke DM (2000). Blockage of drug resistance in vitro by disulfiram, a drug used to treat alcoholism. J Natl Cancer Inst.

[18] Cho HJ, Lee TS, Park JB, Park KK, Choe JY, Sin DI, Park YY, Moon YS, Lee KG, Yeo JH (2007). Disulfiram suppresses invasive ability of osteosarcoma cells via the inhibition of MMP-2 and MMP-9 expression. J Biochem Mol Biol.

[19] Tawari PE, Wang Z, Najlah M, Tsang CW, Kannappan V, Liu P, McConville C, He B, Armesilla AL, Wang W (2015). The cytotoxic mechanisms of disulfiram and copper(II) in cancer cells. Toxicol Res.

[20] Morrison BW, Doudican NA, Patel KR, Orlow SJ (2009). Disulfiram induces copper-dependent stimulation of reactive oxygen species and activation of the extrinsic apoptotic pathway in melanoma. Melanoma Res.

[21] Safi R, Nelson ER, Chitneni SK, Franz KJ, George DJ, Zalutsky MR, McDonnell DP (2014). Copper signaling axis as a target for prostate cancer therapeutics. Cancer Res.

[22] Buac D, Schmitt S, Ventro G, Kona FR, Dou QP (2012). Dithiocarbamate-based coordination compounds as potent proteasome inhibitors in human cancer cells. Mini Rev Med Chem.

[23] Wiggins HL, Wymant JM, Solfa F, Hiscox SE, Taylor KM, Westwell AD, Jones AT (2015). Disulfiram-induced cytotoxicity and endo-lysosomal sequestration of zinc in breast cancer cells. Biochem Pharmacol.

[24] Lewis DJ, Deshmukh P, Tedstone AA, Tuna F, O'Brien P (2014). On the interaction of copper(II) with disulfiram. Chem Commun (Camb).

[25] D'Autreaux B, Toledano MB (2007). ROS as signalling molecules: mechanisms that generate specificity in ROS homeostasis. Nat Rev Mol Cell Biol.

[26] Hart BW, Faiman MD (1993). Bioactivation of S-methyl N,N-diethylthiolcarbamate to S-methyl N,N-diethylthiolcarbamate sulfoxide. Implications for the role of cytochrome P450. Biochem Pharmacol.

[27] Johansson B, Petersen EN, Arnold E (1989). Diethylthiocarbamic acid methyl ester. A potent inhibitor of aldehyde dehydrogenase found in rats treated with disulfiram or diethyldithiocarbamic acid methyl ester. Biochem Pharmacol.

[28] Duan X, Xiao J, Yin Q, Zhang Z, Yu H, Mao S, Li Y (2014). Multi-targeted inhibition of tumor growth and lung metastasis by redox-sensitive shell crosslinked micelles loading disulfiram. Nanotechnology.

[29] Zhang L, Tian B, Li Y, Lei T, Meng J, Yang L, Zhang Y, Chen F, Zhang H, Xu H (2015). A copper-mediated disulfiram-loaded pH-triggered PEG-shedding TAT peptide-modified lipid Nanocapsules for use in tumor therapy. ACS Appl Mater Interfaces.

[30] Song W, Tang Z, Lei T, Wen X, Wang G, Zhang D, Deng M, Tang X, Chen X (2016). Stable loading and delivery of disulfiram with mPEG-PLGA/PCL mixed nanoparticles for tumor therapy. Nanomedicine.

[31] He H, Markoutsa E, Li J, Xu P (2018). Repurposing disulfiram for cancer therapy via targeted nanotechnology through enhanced tumor mass penetration and disassembly. Acta Biomater.

[32] Plumb JA, Milroy R, Kaye SB (1989). Effects of the pH dependence of 3-(4,5-dimethylthiazol-2-yl)-2,5-diphenyl-tetrazolium bromide-formazan absorption on chemosensitivity determined by a novel tetrazolium-based assay. Cancer Res.

[33] Andreottola G, Cadonna M, Foladori P, Gatti G, Lorenzi F, Nardelli P (2007). Heavy metal removal from winery wastewater in the case of restrictive discharge regulation. Water Sci Technol.

[34] Viola-Rhenals M, Patel KR, Jaimes-Santamaria L, Wu G, Liu J, Dou QP (2018). Recent advances in Antabuse (disulfiram): the importance of its metal-binding ability to its anticancer activity. Curr Med Chem.

[35] Cobby J, Mayersohn M, Selliah S (1977). The rapid reduction of disulfiram in blood and plasma. J Pharmacol Exp Ther.

[36] Agarwal RP, McPherson RA, Phillips M (1983). Rapid degradation of disulfiram by serum albumin. Res Commun Chem Pathol Pharmacol.

[37] Madan A, Parkinson A, Faiman MD (1993). Role of flavin-dependent monooxygenases and cytochrome P450 enzymes in the sulfoxidation of S-methyl N,N-diethylthiolcarbamate. Biochem Pharmacol.

[38] Mothes R, Petzold H, Jakob A, Ruffer T, Lang H (2015). Dithiocarbamate copper(I) and silver(I) complexes: synthesis, structure and thermal behavior. Inorg Chim Acta.

[39] Yu Z, Wang F, Milacic V, Li X, Cui QC, Zhang B, Yan B, Dou QP (2007). Evaluation of copper-dependent proteasome-inhibitory and apoptosis-inducing activities of novel pyrrolidine dithiocarbamate analogues. Int J Mol Med.

[40] Nobel CI, Kimland M, Lind B, Orrenius S, Slater AF (1995). Dithiocarbamates induce apoptosis in thymocytes by raising the intracellular level of redox-active copper. J Biol Chem.

[41] Tisato F, Marzano C, Porchia M, Pellei M, Santini C (2010). Copper in diseases and treatments, and copper-based anticancer strategies. Med Res Rev.

[42] Hieger I (1926). The effect of copper compounds upon the growth of carcinoma in the rat. Biochem J.

[43] Johansson B (1992). A review of the pharmacokinetics and pharmacodynamics of disulfiram and its metabolites. Acta Psychiatr Scand Suppl.

