# Supplementary figures and images for: Investigation of the key chemical structures involved in the anticancer activity of disulfiram in A549 non-small cell lung cancer cell line
Source: BMC Cancer. 2018 Jul 21;18:753. doi: 10.1186/s12885-018-4617-x (PMC6054747; doi:10.1186/s12885-018-4617-x)

Suppl 1

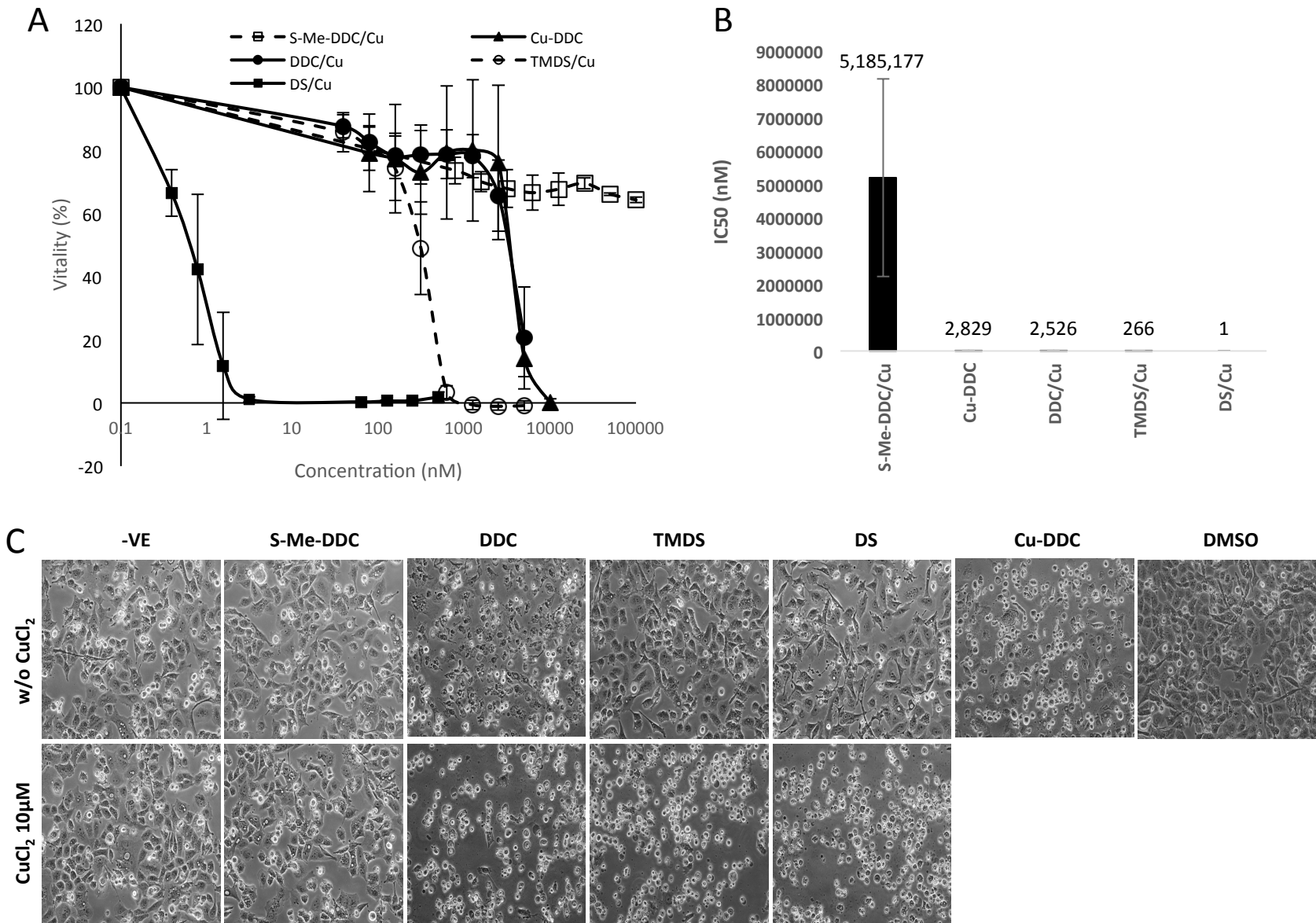

Supplement: Supplementary file 1 — Cytotoxic effect of DS and related compounds on H23 NSCLC cell line. A. (PDF 8177 kb) [file 12885_2018_4617_MOESM1_ESM.pdf]
